# On the impossibility of breaking the echo chamber effect in social media using regulation

**DOI:** 10.1038/s41598-023-50850-6

**Published:** 2024-01-11

**Authors:** Chen Avin, Hadassa Daltrophe, Zvi Lotker

**Affiliations:** 1 Ben-Gurion University of the Negev, Be’er Sheva, Israel; 2 Shamoon College of Engineering, Ashdod, Israel; 3 Bar Ilan University, Ramat Gan, Israel

**Keywords:** Computational science, Computer science, Information technology

## Abstract

As scientists, we are proud of our role in developing the current digital age that enables billions of people to communicate rapidly with others via social media. However, when things go wrong, we are also responsible for taking an ethical stand and trying to solve problems, and this work aims to take a step in this direction. Our goal is to set the foundation for a mathematically formal study of how we might regulate social media and, in particular, address the problem of the echo chamber effect. An echo chamber is a closed system where other voices are excluded by omission, causing your beliefs to become amplified or reinforced. In turn, these bubbles can boost social polarization and extreme political views, and, unfortunately, there is strong evidence that echo chambers exist in social media. The fundamental question we try to answer is: how and can a regulation “break” or reduce the echo chamber effect in social media? Sadly, the paper’s main result is an impossibility result: a general regulation function that achieves this goal (on our social media model) while obeying the core values of democratic societies (freedom of expression and user privacy) does not exist. This result leaves us with hard future choices to make.

## Introduction

Social media (SM) websites and mobile applications have become a significant force driving opinion forming, news consumption, and information sharing among the citizens of our world^1^. As scientists, we are proud of this progress, but when things go wrong^2^, we are also responsible for taking an ethical stand and trying to fix problems^3,4^. This work aims to take a step in this direction. Our goal is to set the foundation for a rigorous study of how we might regulate social media and, in particular, address the problem of the echo chamber effect.

On a basic level, an echo chamber is “an environment in which somebody encounters only opinions and beliefs similar to their own, and does not have to consider alternatives^5^”. By communicating and repeating beliefs within a closed system or network insulated from rebuttal, echo chambers amplify or reinforce beliefs^6,7^. Unfortunately, in recent years, echo chambers have been observed on different social networks, for example, during the 2016 US presidential election^8^ and more recently on controversial topics (e.g., gun control, vaccination, abortion)^9^. In turn, echo chambers are believed to increase social polarization^10,11^, which is getting extreme by opinion amplification^12^. On social media, it was shown that echo chambers limit exposure to diverse perspectives and favor and reinforce presupposed narratives and ideologies^9,13^. However, it is important to note that this view is still under ongoing (academic) debate^14–16^ and we elaborate on this point in the Discussion section.

The crucial problem with echo chambers is that they deprive people (social media users) of a *reality check*, leaving them in a *virtual reality*. In particular, isolation or omission of opinions can hide the truth. However, what is the truth? We leave this out of context for this paper and consider “fact” (following Wittgenstein’s Tractatus^17^) or, in our case, people’s opinions to be the truth. Clearly, avoiding other voices will also prevent exposure to truth or reality. Furthermore, blocking information and voices in democratic states goes against fundamental human rights as expressed in *The Universal Declaration of Human Rights*^18^. In particular, Article 19 describes the *freedom of expression*, freedom of information, and the public’s (and individual) right to know others’ public opinions (among other things).

Motivated by recent debates about social media^19^, specifically, Moshe Vardi’s talk about computer science and ethics^4^, we take the moral viewpoint that echo chambers in social media (and other social interactions) need to be moderated. A critical first observation is that echo chambers have a spectrum that can be quantified. They do not just exist or not exist. We formalize this by considering both the *echo* (bias, reinforcement) and the *chamber* (size) as metrics of an opinion-spreading process. To specify and quatify an *echo chamber effect*, we must, in turn, define a geometry and a frame of reference, as we explain later.

We claim that to reduce the problem of echo chambers in social media, a state *regulation* is in place. Such regulation should not be left to private social media companies, which may have goals and priorities that differ from the well-being of society. Typically, these goals are driven by financial and other interests, and companies may, for example, as been claimed, benefit economically from echo chambers and extreme opinions. In this paper, we consider the social media’s spreading policy (e.g., what posts will appear on a user’s wall or feed) as an “opaque box” that the regulator does not and can not control. We model the regulation as an additional step in the spreading process.

*Paper contribution* We see this paper as a conceptual paper that encourages the community to address the echo chamber effect and other social media-related problems and propose a methodology and initial results.

We first present a general information-spreading model that captures the essence of a social media, friends-based, information-spreading process on a network of users. Our Simple Regulated Social-media Spreading process, denoted as $SRSS$, has several main components: (i) a social network of user and their opinions, (ii) a social media with its spreading policy, (iii) a regulation authority, and (iv) a user reaction function that determines if a user agree, disagree, or ignore a post. A spreading process starts with a user that shares a post and ends with a set of users that received the post and their reaction to it.

In turn, we propose a novel perspective to quantify the echo chamber of a spreading process. We represent an echo chamber with a pair $(\phi , \alpha )$ where $\phi$ is the *echo* that captures the bias, and $\alpha$ is the *chamber* that describes the size of the spreading. To compare different spreading processes, we define an echo chamber metric based on hyperbolic geometry and claim that it obeys two fundamental axioms. We then define the echo chamber effect using a *frame of reference* (i.e., a baseline) for each spreading process.

Next, we discuss and formally define the goals and principles of social media regulation. We set the *goal* of the regulation to “break” the echo-chamber effect and then consider the two most basic principles or values that we want our society and regulation to follow: *freedom of expression* and *user privacy*. Following this formulation, the main result of the paper is, then, an *impossibility result*. For the $SRSS$ process, we prove that any regulation that reduces the echo chamber effect for *any network and social media* must violate either freedom of expression or user privacy for some users.

We complete the technical part by proposing a random linear regulation (RLR) function that preserves the freedom of expression and user privacy and is simple to implement. We conjecture that RLR reduces the echo chamber effect for real-world social media and initially explore it via simulations. We conclude the paper with a discussion that encourages the community to work on these important topics but also presents important objections, limitations, and alternatives to our model and results.

*Background* In their collaborative review^16^, Hadit and Chris studied different aspects of the political dysfunction in social media. In particular, they comprehensively discuss the echo chamber phenomenon and claims of its existence or non-existence^20^ in social networks. Cinelli et al.^9^ studied echo chambers on various social media (Gab, Facebook, Reddit, and Twitter) by measuring network homophily and biased information diffusion. They found dominant homophily interactions and social media segregation. However, the paper didn’t provide a formal definition of the echo-chamber effect, didn’t analyze its size dependence, nor offered a specific metric to measure it. Although Morales et al.^21^ found a platform (Reddit) in which the Echo-Chamber phenomenon is not emphasized in political interactions, they also confirm the existence of similar preferences in other fields.

Filter bubbles^22^ are linked to echo chambers and refer to intellectual isolation caused by algorithms predicting and showing users information they would likely agree with. Examples include Google Personalized Search and Facebook’s personalized feed. Though not in the same formal approach as our paper, efforts to avoid these bubbles using personalized software were addressed in previous works^23,24^. Nguyen^25^ also provided insights on differentiating echo chambers from bubbles. However, the existence of bubbles is under academic discussion, and there are works^26,27^ indicating that the phenomenon is minor or insignificant in terms of news consumption^28,29^, while most people regularly consume content from diverse media^30,31^.

A large-scale recent study of Facebook users in the USA activity from 2020^32–35^ shows that content from ‘like-minded’ sources constitutes most of what people see on the platform. Moreover, reduced exposure to content from like-minded sources increased their exposure to content from cross-cutting sources but did not correspondingly reduce polarization in beliefs or attitudes. Manipulating the users’ feed by omitting reshared posts^33^ or presenting the content in reverse chronological order^34^ did not significantly affect political polarization or attitudes.

While there exists an informal regulation of online content through content moderation policies, community guidelines, and user reporting mechanisms^36^, there is a growing consensus on the need for formal regulation of social media platforms^37^. Still, partisan differences exist regarding the specific issues that regulation should address^37^. The potential benefits of regulation include promoting innovation, increasing competition, and encouraging social media companies to take responsibility for removing harmful content like hate speech and disinformation^38^. However, drawbacks include regulatory burdens that may hinder innovation, challenges in content moderation, and concerns about infringing on free speech values^39^.

In this paper, we present a general information-spreading model that captures the essence of the social media information-spreading process on a network of users. Our model takes inspiration from the fundamental paper of Kempe, Kleinberg & Tardos^40^, which presents and analyses three different, single opinion, spreading models (linear threshold, independent cascade, and weighted cascade) where their goal was to find the subset that maximizes the spread of influence. Subsequent works^41–43^ studied and expanded the spreading model while others^44,45^ examined strategies to contain the misinformation spread by identifying the set of highly influential nodes. In contrast, our model leaves the spreading process of social media as an opaque box and adds (an optional) layer of regulation separately, as described hereafter.

## Social media regulation: model and definitions

In this section, we formally describe the social network model and the *spreading process* of a message (e.g., a post or a tweet) generated by the social media on it. We also introduce into this process the possibility of a *regulator* that can influence the spreading process of social media. We start by defining the network model.

### Definition 1

(Social Network) A social network $N=(G,C)$ is a pair of:

- A graph $G=(V,E)$ such that *V* is a set of *n* nodes (users of the network) and $E \subseteq V \times V$ is a set of undirected edges , represents the connections between the users by which they can share information. For a given network *N*, we denote by *G*(*N*) and *V*(*N*) the graph and the nodes of *N*, respectively.
- A coloring function over the nodes $C:V\rightarrow \{\texttt{red},\texttt{blue} \}$. The different colors represent the variety of opinions of network users. We denote by $\phi ^*=\phi ^*(N)$ the fraction of red users in the network, i.e., $\phi ^*(N) = \frac{|V_{\texttt{red}} |}{|V |}$, where $V_{\texttt{red}}$ is the set of red users. For simplicity, we consider two colors, but the model can support more colors.

We consider the spread of an idea or opinion, represented by a message $m$, in a network *N* and users’ reactions to it. In our model, messages express opinions and are colored with one of the users’ colors. W.l.o.g, we assume that the initial message $m$ is written by a $\texttt{red}$ user *v*; therefore, the message color is also red. Initially, all the users are *inactive*. Formally, each user *u* has a reaction $r(u)$, which is set initially to $r(v)=\texttt{inactive}$.

The spreading process evolves in discrete rounds starting at time $t=0$. Each user has an *inbox* (i.e., Feed) in which the message $m$ can appear while the user is inactive (and overall only once). In turn, each individual *u* who receives a message at time $t-1$ in his *inbox* becomes active at time *t* and must update his reaction $r(u)$ to the message to either $\texttt{agree}$, $\texttt{disagree}$, or $\texttt{ignore}$. For a time *t* let $L_t,D_t$ and $I_t$ denote the set of users which reacted with $\texttt{agree}$, $\texttt{disagree}$, or $\texttt{ignore}$ until time *t*, respectively. Let the *active set* of users at time *t* be $\mathcal {A}_t=\{L_t,D_t,I_t\}$. The set of *inactive* users at time *t* is denoted $\bar{\mathcal {A}}_t = V \setminus \{L_t \cup D_t \cup I_t\}$. The spreading process is non-reversible, namely when a node switches from being inactive to being active, it cannot change its status again during the process; formally, if $v\in L_{t-1}$ then $v\in L_t$, if $v\in D_{t-1}$ then $v\in D_t$ and if $v\in I_{t-1}$ then $v\in I_t$. We can now formally define a *spreading sequence* $\mathcal {P}$ which describe the temporal evolution of the active set $\mathcal {A}_t$,

### Definition 2

(Spreading sequence) Given a network *N* and an initial active set $\mathcal {A}_0=\{L_0=\{v\},D_0=\emptyset ,I_0=\emptyset \}$ such that $C(v)=\texttt{red}$ and $r(v)=\texttt{agree}$, a *spreading sequence* $\mathcal {P}(N,\mathcal {A}_0)$ is a sequence of active sets over time $\{\mathcal {A}_0,\mathcal {A}_1,\ldots \}$ for *N* (describe the users reactions over time). Let $\mathcal {P}_t=\{\mathcal {A}_0,\mathcal {A}_1,\ldots \mathcal {A}_t\}$ denote the spreading sequence up to time *t*.

To generate a social media spreading process, we must define the evolution of the active set from $\mathcal {A}_t$ to $\mathcal {A}_{t+1}$. To do so, we first define what we call *spreading functions*, functions that, given the spreading process history, decide to which users the message will be spread next. We consider two types of spreading functions. The first function $\mathcal {F}_M$ is the *social media spreading function*, that is the algorithm by which the *Social media service* (i.e., Meta, Twitter (now X), etc.) spread a message that a user wants to *“share”* or *“tweet”*. We assume that when an inactive user becomes active and agree an incoming message, the social media service activates its spreading function and recommends *candidates* users to receive the message in the next round (in their inbox). We note that in our model, reacting with agree means also to share the message, whereas, in some Social media services, a separation exists between agree or ‘like’ and ‘share’ reactions.

The second type of spreading function is the *regulation spreading function*, $\mathcal {F}_R$. The regulation function can overwrite the recommendation of the social media spreading function, and it is the final authority that decides in which inboxes of users the message landed at the next time step. We will discuss the principles of regulation functions later. The last component in the spreading process is a user reaction function, $\mathcal {F}_U$, which decides the reaction of users to incoming messages (i.e., agree, disagree or ignore); we will discuss user reaction in more detail shortly. We can now formally describe the evolution of a generic social media spreading process.

**Figure 1:**
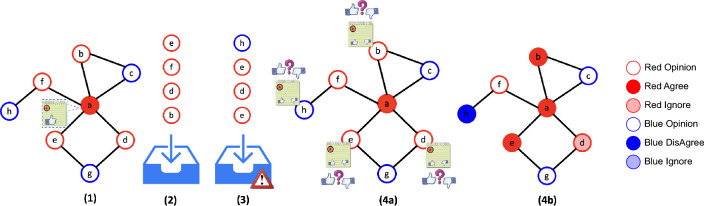
Social media spreading example (Definition 3), on a network $N=(G,C)$. (**1**) Message sharing (e.g., Alice is writing a post). (**2**) Social media candidates for the inbox set (e.g., based on Alice’s neighbors in *G* and their color *C*). (**3**) Regulation decision on the inbox set (e.g., removing f and adding h). (**4a**) Spreading the message to each user in the inbox set and (**4b**) users reaction update (b and e agree the message, d ignore and h disagree).

### Definition 3

(**Social media spreading process**) A social media spreading process evolution is a 5-tuple ${{\,{\textrm{SM}}\,}}(v,N,\mathcal {F}_M,\mathcal {F}_R,\mathcal {F}_U)$ where a single message starts at *v* (with *v*’s color) in a network *N*. The process evolves (and generates a spreading sequence $\mathcal {P}_t=\{\mathcal {A}_0,\mathcal {A}_1,\ldots \mathcal {A}_t\}$) using the social media function $\mathcal {F}_M$, the regulation function $\mathcal {F}_R$ and the user reaction function $\mathcal {F}_U$, in the following order:

1. **Message sharing**: A message *m* is shared by a user at the end of time *t*. A message is shared after a user becomes active and reacts with agree to the message. At the time $t=0$, a single user, *v*, shares its own message.
2. **Social media candidates**: Based on the network *N* and the current process phase $\mathcal {P}_{t}$, the Social Media selects the candidtaes set, ${M}_{t+1}$ using the social media function $\mathcal {F}_M$. ${M}_{t+1}$ is the set candidates: inactive users for which the message *m* will *potentially* (but not necessary) appear on their inbox at time $t+1$. Formally, ${M}_{t+1} = \mathcal {F}_M(N,\mathcal {P}_{t})$.
3. **Regulation**: Based on the network *N*, and the candidtaes set, ${M}_{t+1}$, the regulator determines the *inbox set* of users, ${B}_{t+1}$, using the regulation function $\mathcal {F}_R$. ${B}_{t+1}$ is the set of users for which the message will appear in their inbox at time $t+1$. Formally, ${B}_{t+1} = \mathcal {F}_R(N,{M}_{t+1})$. For the special case of passive regulation (i.e., ${B}_{t+1} = {M}_{t+1}$) we denote $\mathcal {F}_R$ by the identity function, ${{\,\mathrm{\emptyset _R}\,}}$.
4. **User reaction**: To finalize the $t+1$ time step, each inactive user *u* that is exposed to the message *m* in his inbox (feed) at the beginning of the time step $t+1$, reacts to the message with $\texttt{agree},\texttt{disagree}$ or $\texttt{ignore}$, using the user reaction function $\mathcal {F}_U$ and update his status correspondingly. Formally, we activate $\mathcal {F}_U$ for all users in the inbox set, ${B}_{t+1}$, $\forall u \in {B}_{t+1}, ~~ r(u) = \mathcal {F}_U(N,\mathcal {P}_{t},u)$, and $\mathcal {A}_{t+1} = \{L_{t+1}, D_{t+1}, I_{t+1} \}$, such that $L_{t+1}= L_t \cup \{u \mid u \in {B}_{t+1},~r(u) = \texttt{agree} \}$, $D_{t+1} = D_t \cup \{u \mid u \in {B}_{t+1},~r(u) = \texttt{disagree} \}$, $I_{t+1} = I_t \cup \{u \mid u \in {B}_{t+1},~r(u) = \texttt{ignore} \}$.
5. **Repeat or stop**: For every user that updated its reaction to agree, the message is shared at the end of the time step $t+1$ (and we go back to 1). If no new user reacted with agree (i.e., $L_{t+1} = L_t$) the spreading process stops.

Figure 1 visually illustrates steps 1–4 in a social media spreading process evolution as defined in Definition 3.

Note that in the spreading process of Definition 3, the main three functions: the social media function $\mathcal {F}_M$, the regulation function $\mathcal {F}_R$, and the user reaction function $\mathcal {F}_U$ are not defined explicitly, leaving much room for future proposals and research. To realize a concrete spreading process, we first discuss specific models for the social media service spreading policy and for the user reaction behavior. While both processes are complex and not transparent, we next present basic fundamental models for each function.

### Modeling social media spreading function, $\mathcal {F}_M$

Each social media service (e.g., Meta, Twitter) has its internal complex algorithms for deciding how to distribute a message (e.g., post, tweet) that its users share. In general, such a spreading function could depend on the message’s content, each user’s history, and many other factors. Since we study the echo chamber effect (formally defined later) here, we must make some simplifying assumptions. In our model, the spreading is based only on the network’s topology and the users’ coloring. We assume that once shared by user *v*, a message can spread only to neighbors of *v*. Moreover, each node can see a message at most once, namely shared messages at time *t* can appear only in the inbox of inactive users at time $t+1$ (concerning the specific message). We also assume that messages are spread to each neighbor of *v* independently of the other neighbors of *v*. We propose the following *homophily-based* social media spreading function, $\mathcal {F}_M$.

Given a network *N* and the current spreading process at time *t*, $\mathcal {P}_{t}=\{\mathcal {A}_0,\ldots \mathcal {A}_{t}\}$, such that $\mathcal {A}_t=\{L_t,D_t,I_t\}$, let the set of user who changed their status to agree at time *t* denoted by ${\hat{L}}_{t} = L_{t} \setminus L_{t-1}$. Note that ${\hat{L}}_{t}$ is the set of users that do ‘share’ at time *t*. Let $p,q\in [0,1]$ denote two probabilities. The probability that an inactive node $u \in \bar{\mathcal {A}}_{t}$ which is a neighbor of a node $v \in {\hat{L}}_{t}$ will be chosen in the spreading function $\mathcal {F}_M(N,\mathcal {P}_{t-1})$ to receive the message, is defined independently by the function $\delta (u,v)$ as follows:

1 $$\begin{aligned} \delta (v, u)={\left\{ \begin{array}{ll} p, &{} (v, u) \in E, v \in {\hat{L}}_{t}, u \in \bar{\mathcal {A}}_{t}, C(v)=C(u) ~~~~~~\text {(Same color)} \\ q, &{} (v, u) \in E, v \in {\hat{L}}_{t}, u \in \bar{\mathcal {A}}_{t}, C(v) \ne C(u) ~~~~~\text { (Different color)} \\ 0, &{} \text {otherwise} \end{array}\right. } \end{aligned}$$

Formally the social media candidates set ${M}_{t+1} = \mathcal {F}_M(N,\mathcal {P}_{t})$ is selected such for each $(v, u) \in E$, *u* is added to ${M}_{t+1}$, independently, with probability $\delta (u, v)$. In our analysis and simulations, we consider three different settings for $\delta$, defined as follows:

1. *All neighbors* $(p=1,q=1)$: The message spreads to *all* inactive neighbors.
2. *Strong homophily* $(p=1,q=0)$: *The message spreads to all* inactive neighbors *with the same color*.
3. *p*-*homophily* $(p \ge 1/2,q=1-p)$: The message spreading is biased toward inactive neighboring users with the same color but can also reach different color neighbors.

By adjusting *p* and *q* the social media service controls the spreading process. For example, intuitively, Strong homophily will lead to an echo chamber effect since the message will not reach blue users at all. It is suspected that the policies of social media services are homophilic to some extent, where users see more posts similar to their own, even when they have neighbors with other opinions.

### Modeling user reaction function, $\mathcal {F}_U$

When a user receives a new message in his inbox (i.e., feed), how will she react to it? What will cause her to agree or disagree the message? Obviously, this is a non-transparent, complex process that is hard to model exactly. Therefore, we assume for simplicity that the reaction is only based on the number of users who agree or disagree to the message until time *t*, and the color of the user himself when we model the *user reaction function* $\mathcal {F}_U(N,\mathcal {P}_{t},u)$.

More concretely, in this work, we assume that the probability that a user chooses to agree or disagree the message $m$ is proportional to the ratio of agree to the total number of reactions: $\Delta = \frac{|L_t |}{|L_t |+|D_t |}$. In particular, users with different colors react symmetrically but with opposite reactions. Recall that the message color is red. Formally, for each user $u \in {B}_{t+1}$ (the inbox set at time $t+1$), we use the following reaction function:

2 $$\begin{aligned} r(u)=\mathcal {F}_U(N,\mathcal {P}_{t},u)= {\left\{ \begin{array}{ll} \texttt{agree} &{} \text {with probability } \Delta \text { if } C(u)=\texttt{red} \\ \texttt{disagree} &{} \text {with probability } \Delta \text { if } C(u)=\texttt{blue} \\ \texttt{ignore} &{} \text {otherwise} \end{array}\right. } \end{aligned}$$

We say that a user who reacts with agree or disagree *actively reacted* to the message, so a user either actively reacted to a message or ignored it.

The user reaction we consider aims to model the behavior that a red user will more likely support (agree and share) a post with a higher percentage of red votes. When the fraction of blue votes increases, he may be more reluctant and deiced to ignore the message (not share it). On the contrary, a blue user that sees a red post with many red votes will more likely oppose the message by a disagree. Recall that the blue users do *not* share a red message. Many interesting user reaction functions are left for future study, e.g., functions that depend on the number of votes or threshold functions.

Following the previous definitions, in the rest of the paper, we consider a specific model for social media spreading and regulation denoted $SRSS$.

#### Definition 4

[Simple Regulated Social-media Spreading process ($SRSS$)] A simple regulated social-media spreading process ($SRSS$) is a social media spreading process as defined in Definition 3, with a *p*-homophily spreading function $\mathcal {F}_M$ defined by Eq. (1) and a user reaction function $\mathcal {F}_U$ as defined by Eq. (2).

Before continuing with our results, we present several examples of the spreading process.

### Examples of social media spreading process (with passive regulation)

**Figure 2:**
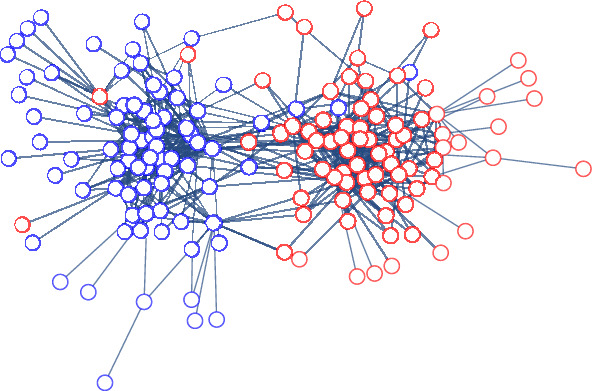
An example network *N*.

For illustration, we next provide a few examples of the social media spreading process, for now, with passive regulation. Figure 2 presents an example network, *N*, with 160 users and 577 edges, for which 50% of the users have red opinion, and 50% have blue opinion (and we use the legend of Figure 1). We will use *N* to demonstrate different social media spreading processes along the paper. We note that *N* is a sampled sub-network from a known political blogosphere network^46^ where each node is associated with a political opinion. As in the original networks, *N* is polarized where most of the edges are within each political group.

**Figure 3:**
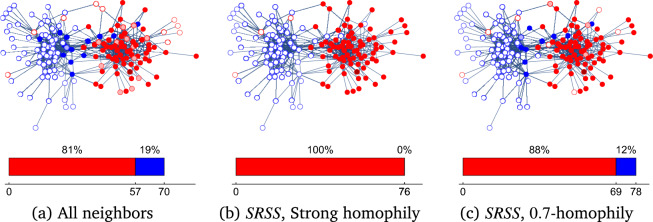
Social media spreading process with passive regulation. Given the network *N* with $\phi ^*(N)= 0.5$, and the user reaction $\mathcal {F}_U$, starting from an arbitrary red node (marked in dark-red), we simulate three spreading functions $\mathcal {F}_M$: (**a**) All neighbors, (**b**) Strong homophily, and (**c**) *p*-homophily with $p=0.7$. We use the legend of Figure 1 to express users’ opinions and reactions. The bar charts beneath each network present the number and fraction of users that *actively reacted* to the original (red) message in the process..

In turn, Figure 3, shows the results of three different social media spreading processes on *N* that start from *v* (denoted in dark red on the figure). The spreading uses passive regulation, and the user reaction function $\mathcal {F}_U$ as defined in Eq. (2). The setup difference between the processes is that each uses a different social media spreading function $\mathcal {F}_M$. We consider three options: (a) ‘All neighbors’, and two versions of the $SRSS$ process: (b) ‘Strong Homophily’, and (c) 0.7-homophily. Each figure presents the states of all users in *N* at the end of the spreading. Beneath it, we show a bar chart with the number and fraction of users that *actively reacted* to the original red message, with agree or disagree.

The first case, with ‘All neighbors’, is shown in Figure 3 (a). In this scenario, all the neighbors of a node who agree to the message (and therefore share it) receive it. As a result, we can observe a diverse spreading. The original message was spread to 96 users, from which 70 actively reacted to the message, 57 red users agree with it and 13 blue users disagree. Additionally, 12 red users and one blue user ignored it. In contrast, the second case, ‘Strong Homophily’, is shown in Figure 3b. Here all the 76 red users belonging to the connected component of *v* received the message. All those users reacted with agree because no blue node received the message. Therefore, only red users actively reacted to the message creating an unbalanced spreading. No active user ignored it. The third case, where the social media spreading function is 0.7-*homophily*, is shown in Figure 3c. This case yields mixed results from the previous two cases. A total of 78 users actively reacted to the message, from which 69 red user agree with it, and 9 blue users disagree. In addition, 3 red users and 2 blue users ignored the message.

Overall we observe that different social media policies can lead to different sets of nodes that actively react to the message. Informally, such policies can clearly lead to *echo chambers* where users are only exposed to similar opinions. In the next section, we discuss how to define and quantity the *echo chamber effect* for social media spreading process.

## The echo chamber effect: a definition

In this section, we propose a methodology to capture the *echo chamber effect* in social media. Echo chambers, informally, limit the exposure of a message to diverse perspectives and favor or reinforce similar narratives^9,32^. It is believed that Echo chambers in social media may increase social and political polarization and extremism, although there are disagreements about this^16^.

Our main observation is that to study the echo chamber effect, it must first be formally quantified in social media networks. To the best of our knowledge, there is no such definition. We propose two qualitative measures to do so.

*(i)* *Metrics*. An echo chamber has two parts, the *echo* of a message spreading, namely the amount by which the spreading is reinforced by similar narratives, and the *chamber* of the spreading, namely the size of the spreading, measured as the number of users who actively reacted to the message.

*(ii)* A *frame of reference* by which the echo chamber of message spreading can be quantified, and a geometry (i.e., distance) to do so. We address these measures in turn.

### Definition 5

(Echo-chamber of a spreading sequence $\mathcal {P}$) Let $\mathcal {P}=\{\mathcal {A}_0,\mathcal {A}_1,\ldots \mathcal {A}_T\}$ be a spreading sequence of a message $m$ starting at a user *v* in a network *N*. Let $\ell =|L_T |$ and $d=|D_T |$ be the number of users that reacted with agree and disagree, respectively, to the message by the end of the spreading sequence $\mathcal {P}$, at time *T*. The *echo-chamber* of $\mathcal {P}$, denoted as $\Psi (\mathcal {P})$, is the vector $(\phi , \alpha )$ such that $\phi =\phi (\Psi )=\frac{\ell }{\ell +d}$ and $\alpha = \alpha (\Psi )= \ell + d$.

We denote $\phi$ as the “echo” of the message spreading, i.e., the fraction of user who actively reacted to the message with agree, and $\alpha$ as the “chamber”, i.e., the size of the message spreading. Next, we consider the frame of reference, which will enable us to compare the echo chambers of two spreadings. First, we define the “distance” between two echo chambers of message-spreading sequences. We would like our distance metric to have the following two axioms, one about echo change and the second about chamber change. We will explain them next.

### Axiom 1

(Echo change) The distance between $(\phi , \alpha )$ and $(\phi \pm \frac{1}{\alpha }, \alpha )$ is constant.

The first axiom states that convincing *one* user (i.e., $\frac{1}{\alpha }$) to change its reaction (from disagree to agree or vice versa) requires a constant effort for every $\phi$ and $\alpha$. Hence, the above distance is constant (for every $\ell$ and *d*).

### Axiom 2

(Chamber change) The distance between $(\phi , \alpha )$ and $(\phi , 2\alpha )$ is constant.

The second axiom states that keeping the same “echo” ($\phi$) but doubling the population requires a constant effort from each user; namely, every user adds a single new user with an opinion (reaction) identical to his. Therefore, the distance should be constant (for every $\ell$ and *d*).

To enable these axioms, we propose to use *hyperbolic geometry*. Recall that the hyperbolic distance (${\text{HD}}$)^47^ between two vectors $\vec {x} =(x_1,x_2)$ and $\vec {y}=(y_1,y_2)$, is defined as ${\text{HD}}(\vec {x},\vec {y})= {{\,{\text{ArcCosh}}\,}}(1+\frac{(x_1-y_1)^2+(x_2-y_2)^2}{2 x_2 y_2}).$ In turn, we use the ${\text{HD}}$ to define the echo-chamber distance between different spreading.

### Definition 6

(Echo-chamber distance) Consider two message spreading processes $\mathcal {P}$ and $\mathcal {P}'$, and their echo chambers $\Psi (\mathcal {P})=(\phi , \alpha )$ and $\Psi (\mathcal {P}')=(\phi ', \alpha ')$. The *echo-chamber distance* between them is the following hyperbolic distance ${{\,\text{ec-dist}\,}}(\Psi (\mathcal {P}), \Psi (\mathcal {P}')) = {\text{HD}}((\phi , \frac{1}{\alpha }),(\phi ', \frac{1}{\alpha '})).$

We can prove the following about the echo-chamber distance (see supplementary material).

### Theorem 1

The echo-chamber distance, as defined in Definition 6, satisfy Axioms 1 and 2.

Until now, we considered a single spreading (i.e., a single starting user and a single message). When we want to quantify the echo-chamber of a network *N* for specific spreading functions and user reaction models, we take the expected spreading of a message. Formally,

### Definition 7

(Echo-chamber of a node and a social media spreading process) For a well-defined social media spreading process *S* (Definition 3), the expected echo-chamber of a node *v* in *S* is defined as the expected echo-chamber of a spreading sequence starting at *v*. The average echo-chamber of *S* is defined as the expected spread of a red message. Formally,

3 $$\begin{aligned} {{\,{\text{EC}}\,}}(S(v)) = {{\,\mathrm{\mathbb {E}}\,}}[\Psi (S(v))] = (\bar{\phi }_v,\bar{\alpha }_v), ~~~ {\text { and }} ~~~ {{\,\text{EC}\,}}(S) = \frac{1}{|V_{\texttt{red}} |}\sum _{v \in V_{\texttt{red}}}{{\,\textrm{EC}\,}}(S(v)) = (\bar{\phi },\bar{\alpha }), \end{aligned}$$

where $\bar{\phi }_v$ and $\bar{\alpha }_v$ are the expected echo and chamber of the spreading process starting at *v*, respectively, and $\bar{\phi }$ and $\bar{\alpha }$ are the expected echo and chamber of the spreading process *S*, respectively.

We finalized our approach by providing for each node, network, and social media spreading process their *points of reference*. In an unbiased world, we would expect that the message reaction (echo) will be similar to the opinions of the general public $\phi ^*(N)$, and we would also like to keep the expected number of active users for a spreading (chamber) the same as it would have been with *a passive* regulation (i.e., no regulation). For a social media spreading process $S = {{\,{\text{SM}}\,}}(v,N,\mathcal {F}_M,\mathcal {F}_R,\mathcal {F}_U)$ we denote by $S_\emptyset$ a similar process but with passive regulation, i.e., $S_\emptyset = {{\,{\text{SM}}\,}}(v,N,\mathcal {F}_M,{{\,\mathrm{\emptyset _R}\,}},\mathcal {F}_U)$. We can now formally define the *reference points* of a node and the spreading process.

### Definition 8

(The reference points for a node and a social media spreading process) For a well defined social media spreading process *S* (Definition 3), the reference point for a node *v* in *S* and the reference point of *S* are defined as,

4 $$\begin{aligned} {{\,\text{Ref}\,}}(S(v)) = (\phi ^*(N),\alpha {{\,\textrm{EC}\,}}(S_\emptyset (v))), \quad {\text { and }}\quad {{\,\text{Ref}\,}}(S) = (\phi ^*(N), \alpha ({{\,\text{EC}\,}}(S_\emptyset )), \end{aligned}$$

where $\phi ^*(N)$ is the echo of the network *N*, namely, the ratio of red users to red or blue users in the coloring of *N*, and $\alpha ({{\,\text{EC}\,}}(S_\emptyset (v))$ and $\alpha ({{\,\text{EC}\,}}(S_\emptyset ))$ are the expected size of active users in the spreading process with passive regulation in $S_\emptyset (v)$ and $S_\emptyset$, respectively.

We define the *echo chamber effect* of a node *v* and a social media process *s*, denoted as ${{\,\text{EC-Effect}\,}}(S(v))$, as the echo-chamber distance between their average echo-chamber and the corresponding reference point. Formally:

### Definition 9

(The Echo-chamber effect of a node and a social media spreading process) The *echo-chamber effect* of a node *v* in a well defined social media spreading process *S* (Definition 3) is the echo-chamber distance between ${{\,\text{EC}\,}}(S(v))$ and its *point of reference* ${{\,\textrm{Ref}\,}}(S(v))$. The *echo-chamber effect* of *S* is the echo-chamber distance between ${{\,\textrm{EC}\,}}(S)$ and its *point of reference* ${{\,\textrm{Ref}\,}}(S)$. Formally,

5 $$\begin{aligned} {{\,\text{EC-Effect}\,}}(S(v)) = {{\,\text{ec-dist}\,}}({{\,\textrm{EC}\,}}(S(v)), {{\,\textrm{Ref}\,}}(S(v))), ~~~~\text { and }~~~~ {{\,\text{EC-Effect}\,}}(S) = {{\,\text{ec-dist}\,}}({{\,\textrm{EC}\,}}(S), {{\,\textrm{Ref}\,}}(S)). \end{aligned}$$

**Figure 4:**
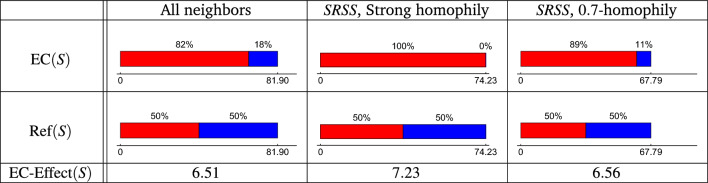
The Echo-chamber (${{\,\textrm{EC}\,}}(S)$), the reference point (${{\,\textrm{Ref}\,}}(S)$) and the Echo-chamber Effect (${{\,\text{EC-Effect}\,}}(S)$) of the three spreading processes of Fig. 3: All neighbors, Strong homophily and 0.7-homophily.

Figure 4 presents the echo-chamber effect of the three social media scenarios we considered in Fig. 3. For each of the three processes, ‘All neighbors’, ‘Strong Homophily’, and ‘0.7-homophily’, we present their echo-chamber (${{\,\textrm{EC}\,}}(S)$), their reference point (${{\,\textrm{Ref}\,}}(S)$) and their echo-chamber effect (${{\,\text{EC-Effect}\,}}(S)$) for the social media.

First, note the (expected) chamber of each process. For ‘All neighbors’, it is 81.90, while for ‘Strong Homophily’ and ‘0.7-homophily’, it is 74.21 and 67.79 nodes, respectively. The echo of the ‘Strong Homophily’ is the strongest. It is 100% since the message reaches only red nodes. Next is ‘0.7-homophily’ with an echo of 89%, and ‘All neighbors’ have the slightest echo of 82%. The reference point for each process (as defined in Eq. (4)) has an echo of 50% (since 50% of the nodes are red and 50% are blue) and the same echo as of the original process (since in this case the process has passive regulation and $S=S_\emptyset$). We can now discuss the echo chamber effect of each process. Recall that the echo chamber effect is defined as the distance between the process’s echo chamber and its reference point. We can observe that the ‘Strong homophily’ has the most significant echo chamber effect, then the 0.7-homophily, and then the ‘All neighbors’ scenario with the smallest echo chamber effect. Recall that all of these scenarios did not include a regulation. In the next section, we discuss desired principles for regulation functions, where our goal is to reduce the echo-chamber effect of a given network.

It is important to note that the geometry selection and the reference point are key components in our methodology. The concrete selection of both determines the echo chamber effect for a given social media spreading process. Therefore it raises the question, what are the correct choices for the reference point and the geometry? The goal of the reference point is to capture a baseline, namely the echo chamber in social media in a “perfect” world. Here, we consider a reference point defined by a hypothetical spreading in a world with passive regulation (i.e., without regulation) but with the same topology and coloring. Future studies can consider cases where the reference point also depends on a modified network topology or an ideal coloring of the users (e.g., random vs. clustered). The goal of the geometry is to quantify the effect. We leave the study of other alternatives of reference points for future work.

## Goals and principles of social media regulation

In the following section, we discuss and formally define which basic properties we would like our regulation functions $\mathcal {F}_R$ to have. We start with the *goal* of the regulation: to “break” the echo-chamber effect. Armed with our formal definitions of a social media spreading process, the echo-chamber effect, and regulation, we can now define what a regulation with a *mitigate outcome* is. Informally, this regulation function always (if possible) reduces the echo-chamber effect and never increases it. Formally,

### Definition 10

(**Mitigate outcome**) Consider a well-defined social media spreading process $S = {{\,\mathrm{\textrm{SM}}\,}}(v,N,\mathcal {F}_M,\mathcal {F}_R,\mathcal {F}_U)$ and its version with passive regulation $S_\emptyset = {{\,\mathrm{\textrm{SM}}\,}}(v,N,\mathcal {F}_M,{{\,\mathrm{\emptyset _R}\,}},\mathcal {F}_U)$. The regulation $\mathcal {F}_R$ has a *mitigate outcome* in *S* if for every node *v* such that its echo-chamber effect with passive regulation, ${{\,\text{EC-Effect}\,}}(S_\emptyset (v))$ is positive, then its echo-chamber effect ${{\,\text{EC-Effect}\,}}(S(v))$ with the regulation $\mathcal {F}_R$ is strictly smaller. Formally,

6 $$\begin{aligned} \forall \;v ~\text {s.t.}~ {{\,\text{EC-Effect}\,}}(S_\emptyset (v))>0: ~~~~~~ {{\,\text{EC-Effect}\,}}(S(v)) < {{\,\text{EC-Effect}\,}}(S_\emptyset (v)) \end{aligned}$$

What can regulation do to eliminate the echo-chamber effect? For example, a trivial regulation could block all posts, empty all feeds, and by that, completely eliminate any echo-chamber effect. This is, of course, an extreme action that gives the regulation an extreme power. Although, by definition, the regulator can intervene in the social media’s spreading process, we may want this intervention to be minimal. Similarly, we need to consider additional ethical issues like privacy (does a regulator need to protect privacy? to what level?) and censorship (to what extent do we allow censorship?)

In this work, we consider the two most basic principles or values that we want our society, and in particular, its regulation system, to follow. These principles, in turn, limit the regulation system’s power. The first principle we consider is *freedom of expression*: An individual’s right to voice their opinions without fear of censorship or legal sanction. We assume that the regulation is *not allowed* to censor any message a user sends; it can only add additional recipients to sent messages. Formally,

### Definition 11

(**Freedom of expression**) A Regulation $\mathcal {F}_R$ has the *freedom of expression* principle when the following holds: the regulation cannot block any recipient to whom the social media indented to deliver the message. Formally, for each time *t* and each user *v*, if the social media decided to add the message to the candidtaes set, i.e., $v \in {M}_t$, then the regulation function must include it in the inbox set of time *t*, ${B}_t$: ${M}_t\subseteq {B}_t$

The second principle we consider is *user privacy*: the regulation does not have access to the user’s opinions (colors), i.e., it cannot use a user’s opinion in making decisions. Formally,

### Definition 12

(**User privacy**) Let *N* and $N'$ denote two networks having the same topology (i.e., graph) but different user coloring. A regulation $\mathcal {F}_R$ has the *user privacy* property when the following holds: For each time *t*, if the candidtaes set, ${M}_t$, is equal in both networks, then for each node *u* the probability that the regulation adds *u* to the inbox set, ${B}_t$, is the same, i.e., independent of the users’ coloring of each network. Formally: if $G(N)=G(N')$ then, $\forall u\in V$, $\mathbb {P}[u\in \mathcal {F}_R(N,{M}_{t})] = \mathbb {P}[u\in \mathcal {F}_R(N',{M}_{t})]$

Note that if two networks have the same graph, i.e., $G(N)=G(N')$, then it implies that they have the same set of users, $V(N)=V(N')$, but not necessarily that their opinions are the same, i.e., $C(N)=C(N')$. This means that the regulation can be topology-dependent, but not coloring-dependent (a stricter version can also assume that the regulation cannot know even the network’s topology). In the next section, we go back to the echo-chamber effect and study the ability of a regulation that has the freedom of expression and user privacy properties to reduce it.

## Breaking the echo-chamber effect: an impossibility result for $SRSS$

In this section, we finally reach the core question of this study: can we use regulation to “break”, or more correctly, to reduce the echo-chamber effect of social media? The paper’s main result is the following impossibility result: regulation cannot have both the *freedom of expression* and *user privacy* properties, **and** have a mitigate outcome for the $SRSS$ model (Definition 4). Formally,

### Theorem 2

(Impossibility) It is impossible for regulation to have both the *freedom of expression* and *user privacy* properties while having a *mitigate outcome* for all simple regulated social-media spreading process, $SRSS$ (Definition 4).

### Proof

The proof is by example. Consider a network *N* of size *n*. The network has *n*/*k* connected components denoted as *islands*. Each island is of size $k=2\log n$ users, it is highly connected, and all users are of the same color. Half of the islands are red, and half are blue; therefore, the network has $50\%$ red users and $50\%$ blue users.

The social media processes *S* employs the $SRSS$. Let *v* be a red node in a small component that starts a spreading process. The expected echo chamber of *v* with *passive regulation*, ${{\,\textrm{EC}\,}}(S_\emptyset (v))$, will be $(\phi , \alpha )=(1, 2 \log n)$ since the message will not leave the connected component and, with high probability, will reach all the nodes in its connected components (formally, this depends on the value of *p* in the spreading function, the “connectivity level” inside the connected component and *n*. We leave the technical details for the full report). By Eq.(4), the reference point for a node *v* in $S_\emptyset$ is therefore ${{\,\textrm{Ref}\,}}(S_\emptyset (v)) =(1/2, 2 \log n)$. So the ${{\,\text{EC-Effect}\,}}(S_\emptyset (v))$ is in turn,

7 $$\begin{aligned} {{\,\text{EC-Effect}\,}}(S_\emptyset (v)) = \textrm{HD}((1, 2 \log n),(1/2, 2 \log n)) ={{\,\textrm{ArcCosh}\,}}(1+\frac{(\frac{1}{2})^2}{2 \frac{1}{4\log ^2 n}}) ={{\,\textrm{ArcCosh}\,}}(1+\frac{1}{2} \log ^2 n) \end{aligned}$$

Recall that we assume that the regulation $\mathcal {F}_R$ holds both the *freedom of expression* and *user privacy* properties. Now assume by contradiction that the regulation also has *mitigate outcome*. This means that $\mathcal {F}_R$ has to spread the message to some blue users outside the connected component of *v*, otherwise the ${{\,\text{EC-Effect}\,}}$ will not decrease. However, since $\mathcal {F}_R$ preserves *user privacy*, namely, it is “color-blinded” if messages arrive at islands of blue nodes, some messages must also arrive at islands of red nodes. In turn, since $\mathcal {F}_R$ preserves *freedom of expression* and has no censorship, the message will spread to all nodes in each red component it arrives at. But, then, by symmetry, messages will spread again (using the regulation) to *new* blue and red islands. The end result of this *birth process* will be with a high probability that a linear fraction of red nodes will receive the message and some blue blue nodes will receive it as well (at most a linear fraction). Therefore the expected echo chamber of *v*, ${{\,\textrm{EC}\,}}(S(v))$ will be, in the best case, (1/2, *cn*), for a constant *c* (independent of *n*). The reference point ${{\,\textrm{Ref}\,}}(S(v))$ is, in fact equal (by definition) to ${{\,\textrm{Ref}\,}}(S_\emptyset (v))$, and therefore equal $(1/2, 2 \log n)$. The ${{\,\text{EC-Effect}\,}}(S(v))$ is, in turn, then

8 $$\begin{aligned} {{\,\text{EC-Effect}\,}}(S(v))&\ge \textrm{HD}((1, \frac{1}{c n}),(1/2, \frac{1}{2 \log n})) \approx {{\,\textrm{ArcCosh}\,}}(1+\frac{(\frac{1}{2})^2}{2 \frac{1}{cn \log n}}) ={{\,\textrm{ArcCosh}\,}}(1+\frac{1}{2} c n \log n). \end{aligned}$$

For each constant *c*, since ${{\,\textrm{ArcCosh}\,}}$ is a monotone unbounded function, there exists then a large enough $n_0$ for which for any $n > n_0$ we have ${{\,\text{EC-Effect}\,}}(S(v)) > {{\,\text{EC-Effect}\,}}(S_\emptyset (v)),$ contradiction to the mitigate outcome of $\mathcal {F}_R$. $\square$

- *Discussion of main result.* Theorem 2 raises some major ethical questions. In particular, what should be the objectives of the regulation function, and at what cost? What are the main tradeoffs (within our context), for example, between freedom of information (to reach outside your bubble) and privacy? Or is censorship unavoidable to reach some goals? Even though some of these questions are philosophical, our community is responsible for modeling and studying them to understand better what we can and cannot do. Avoiding these issues is also taking a stand and, therefore, not an option.

The network topology of the counter-example in the proof of Theorem 2 is far from real-life networks. Therefore, it may be the case that the impossibility result does not hold for networks with some connectivity properties. In the next section, we provide evidence for that and we propose a simple regulation function that may work well in practice.

## Case study: random linear regulation

In this section, we propose *Random Linear Regulation* (RLR), a frame for regulation based on randomness, which complies with the regulation principles mentioned above and manages to reduce the echo chamber for real-world media networks.

**Figure 5:**
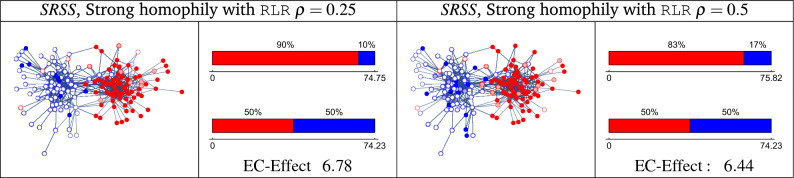
$SRSS$ with Strong Homophily spreading process with RLR. Given the network *N*, $SRSS$ with the ‘Strong Homophily’ spreading function, we simulate the RLR using $\rho =0.25$ and $\rho =0.5$. The network graphs illustrate the users’ opinions and reactions in particular simulations. The Echo-chamber ${{\,\textrm{EC}\,}}(S)$ (the top bar), the reference point ${{\,\textrm{Ref}\,}}(S)$ (the bottom bar), and the Echo-chamber Effect ${{\,\text{EC-Effect}\,}}(S)$ of both spreading processes are presented respectively.

### Definition 13

(Random Linear Regulation) Given the network *N* and the candidtaes set, ${M}_{t}$, the Random Linear Regulation $(\texttt{RLR})$ function with parameter $0 \le \rho \le 1$ is defined as follows: $\mathcal {F}_R= \texttt{RLR} (N,{M}_{t},\rho ) = {M}_{t} \cup R_t$, where $R_t\subseteq V(N)$ is a set of users chosen uniformly at random s.t $|R_t |=\lceil \rho |{M}_{t} | \rceil$. Note that when $\rho =0$, the regulation is passive, i.e., the set $R_t$ is empty.

The first observation about RLR is that it satisfies the two of the regulations’ principles and a third important property, *local proportionality*, which we define next.

### Observation 1

The RLR function satisfies the following principles for each social media it regulates: freedom of expression, privacy-preserving, and local proportionality.

Where the local proportionality property is defined as follows.

### Definition 14

(**Local Proportionality:**) $\mathcal {F}_R$ has the local proportionality property if for each social media, $S = {{\,\mathrm{\textrm{SM}}\,}}(v,N,\mathcal {F}_M,\mathcal {F}_R,\mathcal {F}_U)$, at every time *t*, the number of the additional users that were chosen by the regulation to get the message, (i.e., ${B}_t \setminus {M}_t$), is no more than the number of users that were chosen by the social media to to the candidate set. Formally: $\forall t: |{B}_t \setminus {M}_t |\le |{B}_t\cap {M}_t |$.

The second observation is that RLR is simple to implement, assuming the social media implements it following the regulator’s demand. All it needs is the ability to receive a random user in the system, which is a reasonable requirement. It should also be possible for the regulator to check that RLR was implemented or for social media to provide proof of such implementation.

We know from Theorem 2 that theoretically, the RLR *cannot* preserve freedom of expression and user privacy while having a mitigate outcome. However, in real-world social media (which is extremely different from the isolated example of Theorem 2 proof), we can observe a monotonic reduction in the echo chamber effect as a function of $\rho$, as we following described. Figure 5 demonstrates the use of RLR in the example network we presented in Figure 3b with values of $\rho$, 0.25 and 0.5. The networks in the figure depict the end of the spreading process for particular run examples, while the bars and numbers in the figure presents the average values for 1000 runs of spreading simulations. We can observe in the bars of ${{\,\textrm{EC}\,}}(S)$ that despite the *strong-homophily* behavior of social media, thanks to the RLR spreading, the message has now reached blue users. Furthermore, the network figures demonstrate that, with RLR, there exist red users who have chosen to ignore the message. The chamber of the process with $\texttt{RLR} (\rho =0.25)$ is 74.75, while for $\texttt{RLR} (\rho =0.5)$ it is 75.82 nodes, both are slightly larger than the unregulated value (74.23), albeit not by a significant margin. The echo of RLR with $\rho =0.5$ is $83\%$, which is smaller than the echo of RLR with $\rho =0.25$ ($90\%$) since the message reaches more blue nodes. The reference point for the processes (as defined in Eq. (4)) has an echo and chamber as of the original process (see Figure 3(b)). Finally, the ${{\,\text{EC-Effect}\,}}$ decrease form 7.23 (for the case without regulation) to 6.78 (for $\rho =0.25$) and 6.44 (for $\rho =0.5$) (with $\texttt{RLR}$ regulation). Examining the RLR on larger real networks was also done, as described below.

**Figure 6:**
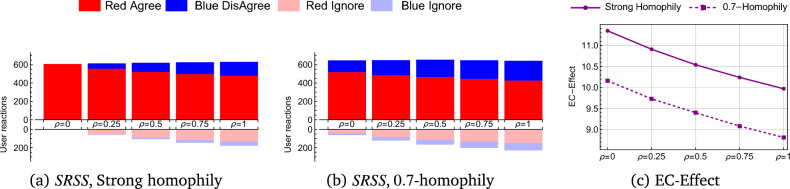
User reactions and EC-effect in the $Bloggers^{52,48}$ graph for the spreading process using RLR regulation. The bar’s height in (**a**) and (**b**) indicate the expectation of 1000 simulations for every $\rho$. The left plot, (**c**), shows the ${{\,\text{EC-Effect}\,}}$ for the ‘Strong homophily’ and ‘0.7-homophily’ social media with different $\rho$.

### Case Study - Experimental Results on a Real Network

Simulations were conducted to study the impact of RLR regulation on a social media platform. The simulations were performed on the $Bloggers^{52,48}$ graph, a network with 1222 users and 16717 edges. This graph represents the largest connected component of a weblog network focused on US politics in 2005^46^. The users are categorized based on their political opinion, with 636 (52%) being conservative (red users) and 586 (48%) being liberal (blue users). The network consists of 7841 red edges, 7301 blue edges, and 1575 green edges, representing links within the respective groups and cross-links (See Figure 1 in supplementary material).

Starting with a randomly chosen red user, we update his status to $\texttt{agree}$ and initiate the spreading process (see Definition 3) using the following parameters: (i) *Spreading function*, $\mathcal {F}_M$ with $\delta$ sets to ‘Strong homophily’ or ‘0.7-homophily’. (ii) RLR regulation with $\rho$ sets to: 0, 0.25, 0.5, 0.75 and 1. After the process is stopped, we count the different reactions to the message for users who were exposed to it. Simulating 1000 times (for each parameter set), we compute the average number of users that reacted with agree, disagree, or ignore as presented in Figure 6.

Figure 6a shows the ‘Strong homophily’ spreading function. When $\rho =0$ (i.e., passive regulation), a red user spreads the message *only* to other red users. Hence, we can see that only red users got the message, which led to only agree response in the population. As expected, increasing the regulation intervention by $\rho$, allows blue users to receive the message and to disagree with it. This, in turn, caused an increase in the amount of the red  ignore (light red), meaning that part of the red users ignored the message and did not share it. Figure 6b for 0.7-*homophily* presents similar results, but in which unanimity of opinion in the red group does not exist in advance (due to the relaxation of the homophily spreading condition). However, in this spreading process, the regulation also has a mitigate outcome on the exposure of information to other parts of the network. Figure 6c demonstrates the changes in ${{\,\text{EC-Effect}\,}}$ for both processes. The regulation had mitigate outcome, which is clearly expressed in the ${{\,\text{EC-Effect}\,}}$ graph behavior: the ${{\,\text{EC-Effect}\,}}$ is monotonically decreasing with the level of regulation (set by $\rho$) during the spreading process.

## Discussion

This article proposes a methodology to study the echo chamber effect within social media and, particularly, how to mitigate it via regulation. We present a formalization of the social media’s spreading process to quantify the effect and, in turn, its mitigation potency. The complexities of modeling the spreading mechanisms within the network present multifaceted challenges, primarily due to the myriad of diverse modalities of social media actions and user responses. We examined a simple spreading model presenting a message only to neighboring users, allowing agree/disagree/ignore responses, but rich enough to capture the essence of the phenomenon. In future work, we would like to consider *dynamic* networks and the case where users’ feeds do not rely on their friends but are based on a recommendation system (e.g., TikTok’s “For You” and Instagram’s “Discover”). In our view, social media regulation is an important research area, currently, in its initial stages. Considerable endeavors are imperative and are required to make progress in solving the variety of problems these platforms present to our society.

*Disclaimer: objections, limitations, and alternatives to our model and results.* There are works^16^ claiming that the very existence of bubbles and the echo chamber is in doubt^26^, where the personalized social media algorithms have a weak impact on content diversity^27^. When tracking individual behaviors, the actual data show that most people take their information primarily from mainstream media and regularly consume content from *diverse* media sources^28,29^. At the same time, fake news website consumption is concentrated among a small group only^30^. Nevertheless, to our understanding, even if the news consumption is diverse in each bubble^31^, the opinion formation about them (due to comments, likes, emotions, etc.) is mainly influenced by the members of your *own* bubble, leading to an echo chamber of *opinions* as we modeled in our work.

We note that our $SRSS$ model assumes a somewhat outdated version of social media since it captures only information received via friends, while modern social media is based on recommendation systems and search engines to receive information. As we demonstrated, echo chambers (e.g., of opinions) can be formed even in our simpler model. It will be interesting to study the problem on more complex models that extend ours, but the impossibility results already hold in a simpler one. Assuming more power to the social media spreading will only make the challenge harder.

Another important concept of our model, which can be criticized, is that the model assumes that social media spreading is an opaque box that cannot be directly regulated. In our model, the regulator can only *modify* the spreading of the social media platform. Moreover, we require that the *regulator* action preserve privacy and freedom of speech regardless of the social media actions. An alternative approach to reduce the echo chamber by the regulator could be to demand transparency or direct oversight of the spreading algorithms of the platform. For example, to require them to eliminate echo chambers by using private information they have. While this may be a legitimate approach, it is more complicated to implement and supervise and does not contradict the fundamental tradeoffs that our work presents.

### Supplementary Information

Supplementary Information.

## Data Availability

The dataset analyzed during the current study is based on the blog network form^46^ and imported using the Mathematica software. The code simulations and further details are available from the corresponding author upon reasonable request.
