# Supplementary material for: On the impossibility of breaking the echo chamber effect in social media using regulation
Source: Sci Rep. 2024 Jan 11;14:1107. doi: 10.1038/s41598-023-50850-6 (PMC10784531; doi:10.1038/s41598-023-50850-6)
Supplement: Supplementary file 1 — Supplementary Information. [file 41598_2023_50850_MOESM1_ESM.pdf]

Supplementary Material

A Proof of Theorem 1

Proof of Theorem 1. Consider the first axiom. Assume that $u,v \geq 0$ and that $u+v \geq 1$. Let $\mathcal{P} = (\frac{u}{u+v}, \frac{1}{u+v})$. When one node changes its opinion from blue to red, we get a new point $\mathcal{P}' = (\frac{u+1}{u+v}, \frac{1}{u+v})$. In the definition of the first axiom, we have ± 1. We note that it is enough to show $+1$ since the distance function is symmetric. Computing the distance $HD(\mathcal{P},\mathcal{P}')$ using hyperbolic distance, it follows that

$$HD(\mathcal{P},\mathcal{P}') = HD(\mathcal{P}',\mathcal{P}) = \text{ArcCosh}[3/2]. \quad (1)$$

Since this do not depend on u,v it follows that the echo-chamber distance satisfy the firs axiom.

We now move to the second axiom. Let $\mathcal{P} = (\frac{u}{u+v}, \frac{1}{u+v})$, $\mathcal{P}' = (\frac{u}{u+v}, \frac{2}{u+v})$ by simple calculation it follows that,

$$HD(\mathcal{P},\mathcal{P}') = HD(\mathcal{P}',\mathcal{P}) = \text{ArcCosh}[5/4]. \quad (2)$$

□

B Figures

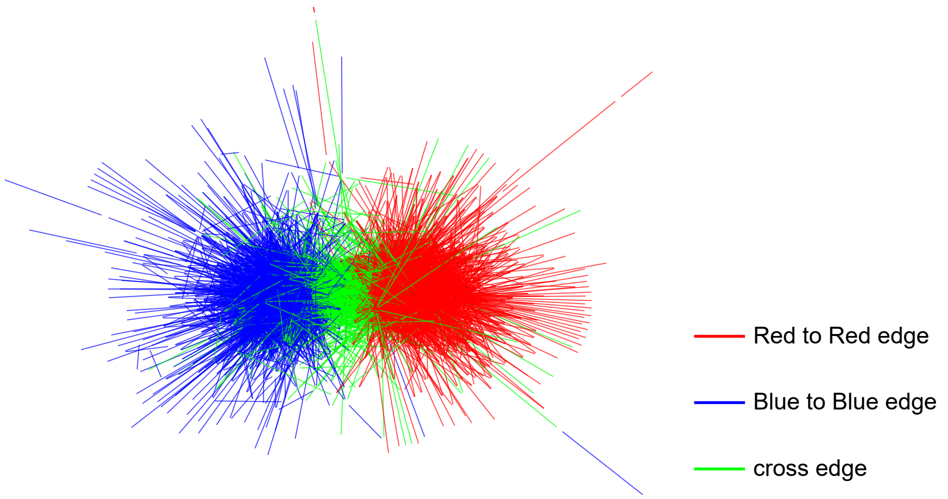

Figure 1. *Bloggers*[52,48] graph. A bi-populated real[1] social network with 52% `red` users and 48% `blue` users. We use this network to simulate the social media spreading process.

References

1. Adamic, L. A. & Glance, N. The political blogosphere and the 2004 us election: divided they blog. In *Proceedings of the 3rd international workshop on Link discovery*, 36–43 (2005).
